# Development and Validation of a Tool to Assess Healthcare Professionals’ Views on Parental Presence During Neonatal Resuscitation

**DOI:** 10.3390/nursrep15100352

**Published:** 2025-09-26

**Authors:** Paraskevi Volaki, Rozeta Sokou, Abraham Pouliakis, Nikoleta Aikaterini Xixi, Zoi Iliodromiti, Styliani Paliatsiou, Georgios Kafalidis, Theodora Boutsikou, Theodoros Xanthos, Nicoletta Iacovidou

**Affiliations:** 1 Neonatal Department, Aretaieio Hospital, School of Medicine, National and Kapodistrian University of Athens, 11528 Athens, Greece; voulavolaki@med.uoa.gr (P.V.); nerinaxixi@med.uoa.gr (N.A.X.); ziliodromiti@med.uoa.gr (Z.I.); stpaliatsiou@med.uoa.gr (S.P.); gkafalidis@gmail.com (G.K.); theobtsk@med.uoa.gr (T.B.); 2 2nd Department of Pathology, National and Kapodistrian University of Athens Medical School, University General Hospital Attikon, 12462 Haidari, Greece; apouliak@med.uoa.gr; 3 School of Health Science, University of West Attica, 12243 Athens, Greece; txanthos@uniwa.gr

**Keywords:** family presence, resuscitation, questionnaire, development, validation

## Abstract

**Background/Objectives**: Childbirth is a natural and joyfully anticipated life event for parents and relatives. Yet, in some cases, it can be a medical emergency requiring immediate intervention, i.e., neonatal resuscitation. The majority of newborns breathe spontaneously; a small number, though, may receive basic life support (assisted transition), and an even smaller but clinically significant number require advanced life support (resuscitation). Within the context of family-centered care, the presence of parents during resuscitation has emerged as a factor with potential implications for emotional adjustment, communication with healthcare providers, and early parent–infant bonding. However, the presence of family members during neonatal resuscitation remains a subject of ongoing debate among healthcare professionals (HCPs). Despite increasing recognition of its potential benefits, HCPs’ views on parental presence during such critical procedures have not been extensively investigated in Greece. This study aims at developing and validating a tool to assess healthcare professionals’ views on parental presence during neonatal resuscitation. **Methods**: A preliminary questionnaire was developed based on the principles of family-centered care and adapted to the Greek population. The first phase included expert assessment of validity, clarity, and relevance using a modified Delphi method. Item Content Validity Index (I-CVI) and Scale CVI (S-CVI) were calculated. Pilot testing was conducted to assess test–retest reliability. Reliability was assessed using the Intraclass Correlation Coefficient (ICC) and Bland–Altman analyses. The study followed the principles of the Declaration of Helsinki, ensuring anonymity, informed consent, and confidentiality. **Results**: The questionnaire includes 37–50 items allocated in four sections. It demonstrated excellent content validity (CVI = 1.00) and good test–retest reliability (ICC = 0.86). Qualitative feedback indicated that the tool is user-friendly and comprehensive. Interestingly, participants expressed genuine concerns regarding the implementation of parental presence in neonatal resuscitation. **Conclusions**: The questionnaire development process led to a comprehensive tool, ready for large-scale testing in order to further establish its validity and internal consistency.

## 1. Introduction

The birth of a child is one of the most joyful and important moments in a family’s life. It marks the beginning of a new era, filled with expectations and hope. This natural process can abruptly become life-threatening and critical for the newborn, and in consequence for the parents, as the need for medical intervention and resuscitation might emerge. In this context, both the actions of HCPs and the degree of parental involvement are both very important.

Childbirth is a milestone not only in the life of the mother but also of her partner, and signifies the beginning if the adjustment to the parental role and the development of an emotional bond with the child. Evidence shows that active partner involvement benefits parents, children, and society, and efforts to encourage such participation, especially during childbirth, have increased over the last decade [1,2,3,4,5,6]. To support partners effectively, HCPs need to understand their experiences and needs, especially in cases where childbirth is complicated. In some countries, such as the United Kingdom, where resuscitation and delivery occur in the same room, partners are usually present during the process [3,7].

In the vast majority, the transition from the intrauterine to the extrauterine life requires minimal action, namely, assessment of the newborn, drying, and thermal control. Only a small number of neonates will need intervention, either simple airway opening maneuvers, the so-called “assisted transition”, or full cardiopulmonary resuscitation [8]. Approximately 85% of newborns will breathe automatically after birth, requiring no further support. An additional 10% will respond to simple stimuli, drying and simple airway management [9]. However, approximately 5% of neonates, which translates into 6–7 million neonates annually worldwide, will need support of their vital functions after birth. Of these, 0.1% of full-term and 10–15% of preterm infants (1.5 to 2.5 million annually) require full cardiopulmonary resuscitation. Fewer than 0.3% need chest compressions, and only 0.05% receive medication, i.e., epinephrine [9].

When the neonate needs special care after birth, this is usually provided on a resuscitaire, usually located near where the delivery occurs. During this critical phase, the parents do not have visual contact with their offspring, and are even kept uninformed with regard to what takes place [10].

Healthcare professionals’ experience from the presence of relatives during resuscitation was studied in different settings, for example, adult or pediatric intensive care units or emergency departments [11,12,13,14]. During the first stages of those studies, HCPs were generally against witnessed resuscitation, expressing concerns about the emotional burden on the families and the risk of physical trauma due to the intense clinical setting, as well as, about the negative effect on the care they provide [13,15,16,17,18]. However, in the ensuing years, those concerns were alleviated. Today, witnessed resuscitation is established in many countries as the standard practice, reflecting the shift of opinion towards a more open and family-centered care [19,20].

From the parental point of view, family presence during resuscitation was studied in depth in adult and pediatric cardiopulmonary resuscitation (CPR), with the results showing that families find it more beneficial for them to witness the resuscitative efforts [10,21,22]. Many parents in the aforementioned studies reported that their presence during pediatric CPR, even simply as observers, contributed to emotional relief, especially in case of a negative outcome [23].

Parental presence during neonatal resuscitation is a strongly debated matter, from which ethical, emotional, and practical questions arise, for parents and HCPs. Including families in this pivotal point in their child’s life is an emerging matter in neonatal care. Understanding the views and opinions of HCPs is a critical step in policy-making that promotes family-centered care and respects parental rights without endangering the quality and effectiveness of medical care. However, data on the effects of family presence during neonatal resuscitation remain scarce. The majority of research focuses on experiences of parents, who were present during the resuscitation of their neonate in the delivery room [24,25].

Regarding HCPs, studies on experiences and perceptions concerning the presence of parents during neonatal life support are limited [3,11,26]. Even more prominent is the gap in the issue in Greek literature, where such studies lack completely. Understanding the HCPs’ point of view plays a crucial role in formulating tangible and realistic health policies that are well adjusted to the needs of the country’s healthcare system, and to its sociocultural background.

Regarding tools designed to assess family-centered care, most focused on adult or pediatric patients [27,28,29]. Recently, Bilal Benbelkheir et al. [30] conducted a systematic review to identify and appraise instruments measuring Family Focused Care in nursing practice. They recommended three tools: the Family Nursing Practice Scale, the Iceland Family Perceived Support Questionnaire, and the Perception of Family Centred Care Staff and Parents questionnaire. However, these tools provide minimal data on parental involvement during critical events such as neonatal resuscitation, highlighting the need for context-specific tools to evaluate healthcare professionals’ perspectives in the neonatal setting.

The main purpose of this study is to develop and validate a tool to assess HCPs’ attitudes and views regarding parental presence during neonatal resuscitation, either immediately after birth or during the infant’s hospitalization in the Neonatal Intensive Care Unit (NICU). Additionally, the questionnaire aims at exploring potential differences in HCPs’ attitudes based on their level of training in neonatal resuscitation, their specialty, and their workplace setting.

## 2. Materials and Methods

### 2.1. Tool Description

We developed the “Questionnaire on the views of HCPs on parental presence during neonatal resuscitation” in order to investigate their opinions, attitudes, and experiences on the presence of one or both parents (or other family members) during resuscitation of neonates, either at the immediate post-partum period or during their hospitalization in the NICUs.

The tool is based on the theory of family-centered care, which promotes and enhances the cooperation between HCPs and parents during resuscitation, and encompasses clinical, ethical, and practical aspects of the matter based on international literature and the specific characteristics of the Greek healthcare system [19,31,32]. Specifically, the tool aims at assessing:

a. The general acceptance of parental presence.
b. The impact on professional practice (team coordination, emotional burden, stress).
c. HCPs’ ethical and legal concerns.
d. Differences in attitudes based on specialty, level of training, workplace setting, and clinical experience.

### 2.2. Questionnaire Design and Development

The development of the questionnaire was based on a combination of existing literature, international guidelines, and empirical observations to secure contextual validity and practical relevance with the Greek healthcare environment.

The development process followed four main stages:

(1) creation of the preliminary version of the questionnaire,
(2) expert panel evaluation,
(3) pilot testing with a representative sample of HCPs, and
(4) evaluation of the questions’ test–retest reliability and clarity.

### 2.3. Ethical Approval and Informed Consent

The study was conducted from June to September 2024, under the principles of the Declaration of Helsinki and its amendments, including anonymity, participation on a voluntary basis, informed consent, and the right to withdraw consent without negative consequences at any time [33]. Participants in both the expert panel and the pilot study were initially informed about the project via email and invited to take part in the expert group or the pilot phase, respectively. Strict confidentiality was maintained at every stage of the study.

The return and completion of the questionnaire were considered an indication of consent to participate. Withdrawal was not possible, as all data were anonymized and participants could not be identified. Participants received information regarding the purpose and nature of the study, potential benefits, and risks. Finally, they were assured that their identity would not be disclosed in the results. The study protocol was approved by the by the Institutional Review Board of Aretaieio Hospital, School of Medicine, National and Kapodistrian University of Athens (protocol code 596; approval date: 14 May 2024).

### 2.4. Content Validity Testing

The Delphi method is suitable for the systematic collection of expert opinions and includes distinct stages [34,35]:

(1) selection of experts,
(2) first round of evaluation, where experts assess the questions,
(3) feedback and re-evaluation,
(4) achievement of consensus.

In this study, a modified version of the method was used; initially an expert panel was created, and in the first round of evaluation, the experts completed a content evaluation form and provided comments. Then, feedback was given without presenting the agreement indices, and finally, the final version of the questionnaire was constructed by the principal investigator, taking also in consideration the experts’ group comments. According to Keeney et al. [34], as experts we defined “informed individuals”, “specialists in their field”, and “people with knowledge related to neonatal resuscitation”. Each expert received a supplementary letter that described the objective of the tool and requested the expert’s evaluation on the tool’s content, relevance, and clarity. Each evaluator assessed the validity of each individual item and was invited to comment on its clarity, relevance, representativeness, and difficulty within the context of the section to which it belonged. To estimate the content validity of the questionnaire, the Content Validity Index (CVI) was used. The panel of experts rated the clarity and relevance of each item using a 4-point Likert scale (1 = not relevant to 4 = highly relevant). The item-level CVI (I-CVI) was calculated as the proportion of experts who rated the item as 3 or 4, divided by the total number of experts. Values of I-CVI ≥ 0.78 were considered as acceptable. The average scale-level CVI (S-CVI/Ave) was calculated to estimate the overall content validity of the tool [36]. In addition, they were encouraged to provide feedback on the overall formatting and contextual relevance of the tool, as well as to suggest any changes or improvements they deemed appropriate. Finally, after repeated rounds of opinion exchange, the final form of the questionnaire was established.

### 2.5. Pilot Study in a Small Representative Sample of HCPs and Test–Retest Reliability

To assess the degree of clarity, understanding, and functionality of the questions, a small-scale pilot test of the tool was conducted using the questionnaire as it was formulated after the first phase of expert evaluation. In this pilot study, healthcare professionals from various professional and educational backgrounds were included. All participants were selected from the Hellenic Society of Cardiopulmonary Resuscitation based on their availability and willingness to participate.

To assess the reliability of responses, that is, how consistently the questionnaire captures the same characteristics when completed by the same individuals after a short period, the test–retest method was applied. The following steps were followed [37]:

(1) Initial Administration (Test): The questionnaire was distributed to a representative sample of participants.
(2) Waiting Interval: A sufficient time gap was allowed between administrations to avoid “mechanical” recall of previous answers, but not so long that the underlying construct being measured could genuinely change. This interval should be more than two weeks but less than six, with flexibility depending on the nature of the questionnaire.
(3) Second Administration (Retest): The same questionnaire was administered again to the same participants.

### 2.6. Statistical Analysis

To assess the agreement between the two tests, the Intraclass Correlation Coefficient (ICC) and the corresponding 95% confidence interval were used. The interpretation of ICC values if < 0.50 denotes poor reliability, 0.50–0.7 moderate reliability, 0.75–0.90 good reliability, and > 0.90 excellent reliability. The calculation of the ICC in the study was based on a two-way mixed-effects model, with fixed raters and absolute agreement [38]. The calculations were performed using the icc() function from the psych package in R [39,40].

Additionally, to evaluate the agreement of responses between the two measurements for each question, a Bland–Altman plot was constructed. The Bland–Altman method analyzes the differences between the two measurements in relation to their mean. These plots include [41]:

a. *x*-axis: the mean value of the two responses before and after,
b. *y*-axis: the difference between the responses at the two time points,
c. A horizontal blue line at the height of the mean difference, indicating the systematic difference (bias) between the two methods,
d. Two dashed red lines representing the confidence intervals. Approximately 95% of the differences are expected to lie within these limits; large deviations from these limits indicate cases where the measurements significantly disagree.

Data were collected in excel data sheets and subsequently statistical processing was performed within the environment of the R language (version 4.5.1) [39].

## 3. Results

### 3.1. Questionnaire Development

The initial questionnaire included 17 core items, which were designed in accordance with the international guidelines of the American Heart Association (AHA), the American Academy of Pediatrics (AAP) [42], and the European Resuscitation Council (ERC) [9]. Additionally, outcomes of previously published studies on family-centered care and witnessed resuscitation were used [9,18,19], along with real-world clinical data from obstetrics and neonatal practices in Greece. Some items were adjusted from already published tools [3,11,26], whereas others were fully redesigned to reflect the distinct cultural and structural features of the Greek context.

The questionnaire consists of four sections, with a set of common questions posed to all participants. The total number of items ranges from 37 to 54, depending on the answers to filter questions, based on a conditional logic flow implemented via the Google Forms platform. Most questions use a five-point Likert scale (1 = Strongly Disagree to 5 = Strongly Agree). Others are dichotomous (Yes/No) or multiple choice (in demographic questions).

### 3.2. Questionnaire Structure

#### 3.2.1. Demographics and Population Characteristics

This section aimed at collecting data of the participants’ general characteristics, namely gender, age, profession, educational background, years of previous clinical experience, religious beliefs, and previous Newborn Life Support (NLS) training. It consisted of 14 core questions that all participants had to answer. The questionnaire was implemented using Google Forms and incorporated conditional logic. If a participant indicated that she/he had children, she/he was automatically redirected to an additional sub-section with three targeted questions. These questions concerned personal experience of resuscitation of their child (if applicable), as well as their desire or attitude toward being present during neonatal resuscitation for their child, under similar clinical circumstances.

##### Workplace-Dependent Questions

Participants were automatically led to the respective subsection, depending on their workplace (maternity ward, neonatal unit, other hospital setting, non-active HCPs). Each section investigates specific aspects, including:

a. The frequency of participation in neonatal resuscitation.
b. The perceived feasibility of implementing family presence in this specific environment
c. The degree of professional coordination, the intra-group support, and the relative organization mindset.

##### Core Questions

The questionnaire includes 17 core questions addressed to all participants, focusing on the right and feasibility of parental presence and physical proximity during neonatal resuscitation. These questions investigate the possibility of parental trauma, stress, emotional burden, and at the same time, the degree of efficacy and coordination of the medical team, as well as any inconvenience that may be imposed on the team by the presence of the family. Both early and late parental involvement were assessed.

These core questions investigate the following aspects:

a. Personal preference regarding parental presence during resuscitation.
b. Perceived impact on efficacy of care, team coordination, and emotional burden of HCPs.
c. The possible legal and ethical concerns that may arise.
d. The perceived parental psychological outcomes (i.e., trauma, stress, guilt, long-term emotional distress) from their exposure to the process of resuscitation.

### 3.3. Content Validity Test

The content validity of the questionnaire was assessed using the Content Validity Index (CVI). All items received an I-CVI score of 1.00, indicating perfect agreement among experts regarding the relevance of each item. Additionally, the scale-level CVI (S-CVI) was calculated using the average method (S-CVI/Ave), which also achieved a score of 1.00. This result reflects excellent overall content validity, confirming that the tool was consistently given good expert ratings across all its items.

Supplementary to the CVI assessment, expert feedback was equally encouraging. The main point of interest was the refinement of the wording of several questions for improved clarity and inclusion of neutral response options such as “neither agree nor disagree” where appropriate. Structural reorganization of the questionnaire flow to optimize user experience (e.g., when delivered via Google Forms) was also suggested.

### 3.4. Test–Retest Reliability

The test–retest reliability of the questionnaire was evaluated with readministration of the tool to 20 HCPs representing various healthcare sectors and training levels (e.g., physicians, midwives, NLS-trained and untrained). The questionnaire was administered twice, with a time interval greater than two weeks but less than of six weeks between each distribution. Semi-structured interviews were also conducted to explore participants’ experience and understanding of the tool.

The sample included 19 women (95%) and 1 man. They were randomly selected, and were all members of the Hellenic Society of Cardiopulmonary Resuscitation based on their availability and willingness to participate; this stage of the study requires a small sample. All members of this society were eligible to participate as they are actively involved in neonatal resuscitation. Of the participants, 11 were midwives (55%) and 9 were physicians (45%), of whom 6 (66.7%) were in NLS training. Eight participants (40%) were parents; among them, 3 (37.5%) had experienced neonatal resuscitation involving their own child.

Only one parent (12.5%) had been present during a neonatal resuscitation; among the others, one would have preferred to be present, while the other would not. The majority (>50%) were employed in maternity wards.

The questionnaire was primarily evaluated for reliability on the final subset of questions concerning attitudes and opinions toward parental presence during neonatal resuscitation, which constitutes the main focus of the study. The analysis demonstrated good overall test–retest reliability, with a mean intraclass correlation coefficient (ICC) of 0.86 (±0.06). Individual ICCs were calculated for each item (see Table 1), revealing excellent reliability for three items (>0.90), good reliability (>0.75) for fourteen items, and moderate reliability (0.73) for one item. These results support the tool’s adequacy for broader application in larger samples.

**Figure 1:**
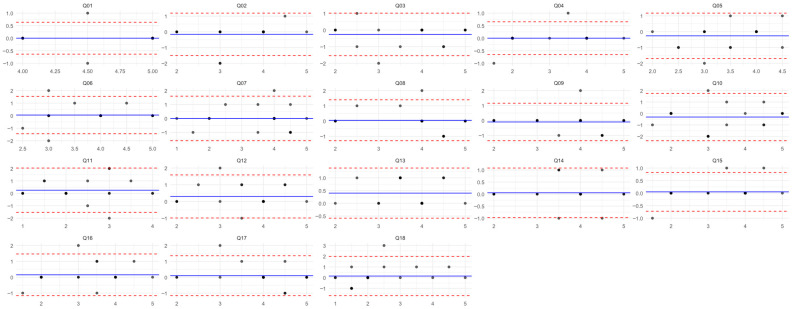
Bland–Altman plot for 18 questionnaire items. See Section 2.6 for interpretation methodology.

The analysis of the Bland–Altman plot (Figure 1) indicates no systematic bias in the differences between responses, and for most items there is no consistent variation between the two measurement time points. For all items, the 95% confidence interval (CI) is approximately 2–3 Likert-scale points, which is considered an acceptable range for assessing test–retest reliability in psychometric instruments. The distribution of the differences around the zero line, without a clear upward or downward trend, suggests the absence of proportional bias, meaning the differences do not increase as the value of the response increases. The majority of the differences lie between −1 and +1 (i.e., variations of only one Likert point), which reflects a very good level of agreement, especially considering the Likert-type format of the questionnaire. It was very rare for the same individual to respond with a variation of 2 or more scale points.

### 3.5. Qualitative Assessment of Semi-Structured Interviews

The semi-structured interviews focused on three main areas:

(1) Completion time
(2) Difficulty
(3) Overall feedback

The mean time needed to answer all questions varied from 10 to 15 min. Participants found the tool “comprehensive” and “concise”, while they mentioned that the use of Google Forms helped answer the questions faster from a computer or a mobile phone. Participants did not report any difficulty in understanding the questionnaire and noted that it was well-structured and user-friendly. Due to minor technical issues that occurred on mobile devices (e.g., hidden questions during scrolling), modifications were made to the structure of the Google Form.

In addition, participants were asked to provide their overall impression of the questionnaire and its subject matter. The wording of the questions did not seem to be of concern to them as much as the topic under investigation. Most responses expressed the participants’ concerns about whether a change in current practice regarding witnessed resuscitation is realistically feasible in the Greek clinical setting, and several noted that the questionnaire was an opportunity for personal reflection. Interestingly, nearly all participants expressed a desire to learn the results of the study and the views of other HCPs on the subject. Three participants mentioned that if they placed themselves in the role of the parent, their answers would have been different from those they gave as HCPs.

## 4. Discussion

The development and validation of a tool to study HCPs’ attitudes and views regarding parental presence during neonatal resuscitation was deemed necessary, as existing literature underscores the multifactorial nature of the matter and the lack of widely accepted clinical guidelines in many healthcare systems [43]. The form of the questionnaire reflects the need for a holistic approach to the matter, while each section was formed per evidence-based and empirical data [10,21,22]. The current study represents an early validation stage of this tool.

At first, a detailed demographics section (gender, age, years of professional experience, academic qualifications, area of specialization, and participation in educational or training seminars) was included to highlight the effect of these factors on the views and attitudes of HCPs. Academic qualifications and formal training seem to play an important role in shaping attitudes regarding the presence of the family during the resuscitation process [44,45]. Several studies from around the globe, support that training seminars, exposure to modern research data, and policies lead to a more positive attitude towards witnessed resuscitation [46,47,48,49]. Professionals who received training or had attended related workshops appear to have a better understanding of how beneficial parental presence can be, for the family and for the provision of care, and are willing to support it [46,47]. Studies on emergency department physicians in French hospitals showed that younger doctors with previous training on family handling during resuscitation are more prone to accept this practice and implement it, as opposed to nurses [48,49]. This comes to further underline the need for the questionnaire to investigate, within the demographic section, whether participants had attended a neonatal resuscitation training seminar. This parameter does not just record training status, but also allows for the exploration of a possible association between education and HCPs’ attitudes. In this way, it increases the interpretive value of the study’s findings.

Parental status of the participants also constitutes an important variable that affects their views on family presence during resuscitation, which was also pointed out by the participants of this pilot study during the semi-structured interviews. The participants, who had personal experiences as parents, are more likely to have a deeper understanding of the emotional needs and preferences of parents during these critical moments for their child’s life. This stronger empathy in this case can lead to a more favorable attitude towards parental presence, not merely as a preference but rather as an established right related to transparency in family-centered care. At the same time, their parental role may influence how they assess the psychological impact of parental presence or absence, as well as the support needs that arise in case of resuscitation. Of equal importance is the personal experience that some HCPs may have had with the resuscitation of their children. Such an experience can profoundly affect their perception of the value of parental presence during neonatal resuscitation. Having themselves been in the position of the parent, their understanding of the emotional burden, the need for visibility during interventions, and the relief that comes from being informed and involved is greatly increased. Studies, such as that of Mian et al. (2007), showed that exposure to real-life Family-Witnessed Resuscitation (FWR) events, whether as professionals or as parents, creates a positive attitude toward this practice, as it increases empathy and an understanding of the psychological needs of the family during such critical moments [50]. It is worth noting that in the present study, three participants remarked that their responses might have differed had they approached the questions from the perspective of a parent rather than a healthcare provider.

Additionally, involvement, under any role, in similar situations can gravely influence the HCPs’ point of view. In the meta-analysis of Oczkowski et al. (2015), it was shown that personal experiences and involvement in resuscitation settings can enhance the perception that parental presence ensures transparency, improves communication with the healthcare team, and alleviates psychological distress in cases of loss [51]. This empirical dimension reinforces the importance of a special subsection in the questionnaire that investigates personal experience, to better interpret the views and attitudes of the participants.

Various studies in HCPs cohorts demonstrated that the more experienced professionals are in CPR, the more likely they are to have witnessed or participated in FWR [46]. This tendency is more pronounced among workers in high-exposure clinical settings (i.e., in the emergency department and intensive care units). Moreover, as previously mentioned, it appears that HCPs with prior experience in FWR tend to be more supportive of this practice, not only as an option that could be offered to the family, but also as a right of the patient and their relatives [50,52]. With that under consideration, the questionnaire was formulated in such a manner as to reflect more precisely the unique characteristics of each group of professionals. Specifically, the questions were organized using conditional logic in Google Forms, allowing participants to respond to specific sections that better suited their professional role and workplace setting (e.g., delivery room, Neonatal Intensive Care Unit, Emergency Department, etc.). This approach was necessary, as experience, frequency of involvement in resuscitation events, and team dynamics vary significantly across different clinical settings. For example, delivery room or NICU personnel are more experienced than pediatricians of the private sector, furthermore, different settings allow for different degrees of parental involvement. Through this targeted structure, the questionnaire aims at drawing safe conclusions by providing representative data facilitating the investigation of various work environment-dependent factors.

After the first demographic section, the ensuing ones and their subsections reflect the basic axes recognized in the international literature as defining factors that either facilitate or obstruct the implication of FWR [53,54].

Directly investigating whether HCPs accept or reject the presence of parents during assisted transition or resuscitation is fundamental to mapping their attitudes. This is particularly important, as research indicates that a positive attitude towards FWR is reinforced through clinical experience or appropriate training. Therefore, documenting these attitudes is essential for the development of targeted educational interventions [50,52]. The questions that address the perceived impact on the effectiveness of care, the coordination of the team, and on the emotional burden on HCPs are based on empirical data that highlight the practical challenges of FWR implication. Healthcare professionals often express the fear that family presence can undermine the efficacy, safety, and coherence of the medical team during an emergency intervention [53]. However, in environments where there is adherence to appropriate protocols and sufficient support from facilitators or family “mediators”, the obstacles seem to be significantly less [52].

The inclusion of questions addressing legal and ethical concerns in our questionnaire is justified by the fact that fears consistently emerge in the literature as inhibiting factors in supporting FWR. Studies showed that HCPs worry about the possibility of medical actions being misinterpreted by parents, the risk of legal consequences in case of an adverse event, and the ethical dilemma between the need to focus on medical care and the parents’ desire to be present [53,54]. These questions aim at revealing whether such concerns are common among HCPs, and if so, to investigate if they are associated with parameters like previous experience, education, and work environment.

Questions related to the psychological consequences for parents (such as the possibility of trauma, anxiety, or long-term emotional burden), as expressed by HCPs, were also added, as some studies report HCPs’ reservations regarding parents’ mental resilience during moments of intense stress [53]. Family presence can also be beneficial, positively affecting medical performance and helping with acceptance and coping, especially when the relatives are supported by properly trained professionals [55].

One aspect of the questionnaire addresses HCPs’ beliefs regarding the importance of protocols and appropriate team training to support the presence of family members during neonatal resuscitation and related procedures. This thematic section aims at capturing the extent to which HCPs consider this framework essential for the safe and smooth transition of parents into the resuscitation setting. Ferreira et al. [56] reported that the majority of HCPs believe that health facilities should have written protocols and training programs regarding the implementation of FWR in order to secure the efficiency of the resuscitation team members and the safety of both the patients and their relatives.

The international literature highlights the need for clear and universally accepted guidelines (i.e., the designation of a professional in charge (a) of exclusively accompanying the parents throughout the entire resuscitation process, (b) of the support of the family’s psychosocial needs in order to prevent traumatic experiences or negative memories, and (c) of the prior assessment of the family’s emotional reactions to prevent interference with care) to carry out a successful FWR in the clinical setting [53,54,57,58]. Regardless of the gradual recognition that FWR has gained, a significant number of HCPs insist on their negative attitude towards this practice. The commonest concern reflects the fear of emotional burden, the possible hamper of medical care, and the loss of concentration among team members, factors that also raise the risk of legal consequences. Additionally, practical matters are also a concern, namely the existence of an appropriate space for family support [53,57,58]. These beliefs justify the incorporation of related sections into the questionnaire to better investigate the effect, in order to capture personal perceptions and the perceived impact of parental presence on clinical practice. The inclusion of these questions in our tool not only allows for the evaluation of the existing institutional framework, but also for the identification of areas for intervention for the development of policies and educational practices that will make parental presence sustainable, safe, and beneficial.

Another important aspect is the distinction between the initial phase of resuscitation and prolonged resuscitation. Many studies recognize the defining role of duration on the attitudes of the involved personnel. HCPs appear to be more positive to FWR during the initial phase of resuscitation, which involves less intense interventions, such as tactile stimuli and inflation breaths, while they remain skeptical when it comes to advanced resuscitation, which includes invasive interventions like intubation and drug administration [51,59]. These concerns are associated with the intensity of the situation, the risk of complications, and the emotional burden placed on the relatives who are present. At the same time, prolonged resuscitation increases the likelihood of an adverse outcome, which further intensifies HCPs’ ethical and legal concerns [53,54]. For this reason, the distinction of questions in the questionnaire based on the phase of resuscitation was essential to reveal variations in HCPs’ views depending on the intensity of the interventions.

The test–retest analysis confirmed satisfactory overall reliability (mean ICC = 0.86), with most items demonstrating good to excellent reliability. The only item that showed slightly lower agreement at retest concerned whether witnessing resuscitation could be traumatic for parents. This result may be explained by the fact that individuals often change their views over time, and under different circumstances and emotional status.

The semi-structured interviews demonstrated that participants evaluated the questionnaire favorably, characterizing it as comprehensive and clinically meaningful. No concerns were raised regarding item wording; however, the principal issue identified was the perceived feasibility of implementing witnessed neonatal resuscitation within the Greek healthcare context. Furthermore, participants expressed strong interest in the study’s outcomes and valued the opportunity for professional self-reflection, thereby reinforcing the instrument’s relevance, validity, and potential utility. All these findings indicate that the instrument demonstrates initial reliability and validity, yet it should be interpreted within the context of an early-stage validation study with a limited sample. Further application of the tool in a larger study, as part of the validation process to assess HCPs’ beliefs regarding family presence during neonatal resuscitation, will enable an in-depth investigation of such findings, potentially shed light on improvements in clinical practice in Greece, and strengthen efforts to address reservations or frequent changes of opinion regarding family presence during neonatal resuscitation, by highlighting the potential benefits of this practice. This process will facilitate a deeper understanding of HCPs’ attitudes, confirm the appropriateness of the content for the target population, and enhance the ability to design interventions that promote family-centered care. The proposed tool, once applied in the Greek population, has the potential to contribute to the identification of HCPs’ beliefs about the events under study, highlight aspects that should be considered by the healthcare team when engaging the family as a partner in neonatal care, and ultimately improve the quality of care in this context. Furthermore, this tool could be adapted for use in other national contexts, taking in consideration the cultural diversity of each setting. Ferreira et al. (2018) in a study in Brazil with a larger sample, identified key domains including the benefits of and strategies for family integration into the care process [56]. Comparing our findings with such international experiences reinforces the notion that the present study is part of a broader and growing global discourse.

Although several instruments exist to assess family-centered care, they provide limited information on parental involvement during critical events such as neonatal resuscitation, highlighting the need for context-specific tools to evaluate HCPs’ perspectives in the neonatal setting [30,60,61]. Dall’Oglio et al. [29] adapted the Family-Centred Care Questionnaire-Revised (FCCQ-R) for Italian NICUs, and modified items to reflect NICU-specific procedures, resulting in the FCCQ-R@it-NICU. This tool proved to be valid and reliable, and captured staff perceptions of current and necessary practices according to the FCC framework. Work experience, education, and professional role influenced staff perceptions, and the involvement of experienced NICU professionals was essential for validation. The FCCQ-R@it-NICU enables different stakeholders to improve the quality and humanization of care, and underscores the importance of developing context-specific instruments, as in the present study, to assess HCPs’ views on parental presence during neonatal resuscitation and support evidence-based improvements in clinical practice.

This study has limitations. First, the sample size, although it corresponds to the minimum acceptable ratio of participants to items for the pilot phase of questionnaire development, it is relatively small (40 questionnaires, 20 participants) and limits generalizability. Additionally, there is scarcity of data in Greek and international literature, and absence of universally accepted guidelines on FWR. The above-mentioned made the development of an appropriate tool even more challenging, while also highlighting the need for further dialogue and research support in this specific field.

It is important to emphasize that the present study is a pilot investigation aiming at validating this tool with a relatively small sample size of 20 participants, representing an initial step toward a more comprehensive research program. While test–retest reliability and content validity were assessed, internal consistency analyses (e.g., Cronbach’s alpha) were not performed at this stage. This pilot phase allows us to preliminarily assess the tool’s feasibility, relevance, and reliability. To better establish the robustness of the tool and its construct validity, a large-scale analysis will be undertaken to assess aspects like internal consistency. Furthermore, after establishing the suitability of the dataset (e.g., Kaiser Meyer Olkin test, Bartlett’s test), exploratory factor analyses will be conducted to locate the underlying dimensions and structure of the questionnaire [62]. This work represents an early validation stage of this tool-phase one of a larger, ongoing effort to refine and validate the tool, ultimately contributing to evidence-based improvements in clinical practice regarding family presence during neonatal resuscitation.

## 5. Conclusions

The present study contributed to the development and pilot evaluation of a questionnaire that shows HCPs’ beliefs and attitudes regarding parental presence during neonatal resuscitation, adapted to the specific cultural and clinical context of Greece. The tool demonstrated satisfactory statistical properties, allowing for the reliable assessment of relevant perceptions.

It is worth mentioning that, for the first time in Greek settings, such a tool has been created, which is structured and psychometrically validated and can be used for research purposes and as a self-assessment and educational support instrument for HCPs. The use of this questionnaire enables the understanding of the factors affecting HCPs’ views, educational needs, and the need for the implementation of policies on the family-centered care model in the neonatal units. In conclusion, this study constitutes an important first step in the establishment of policies and practices that reinforce the cooperation among HCPs and families during these critical moments in neonatal care.

## Figures and Tables

**Table 1:** Intraclass Correlation Coefficients (ICCs) and corresponding 95% Confidence Intervals (CIs) for each question of the assessment tool.

Question	ICC	95% CI
Q01: Parents should be informed antenatally about the possibility of neonatal resuscitation at birth.	0.89	0.73–0.96
Q02: Parents have the right to be present during resuscitation.	0.84	0.59–0.94
Q03: Parents should be allowed to be present during the resuscitation of their newborn if they wish.	0.90	0.74–0.96
Q04: Parents should be able to see or touch their baby during resuscitation.	0.98	0.94–0.99
Q05: Witnessing a resuscitation is a traumatic experience for parents.	0.73	0.32–0.89
Q06: The parents’ wish to be present should be documented in the unit’s policy.	0.77	0.42–0.91
Q07: The effectiveness of HCPs is affected by the parents’ presence.	0.88	0.70–0.95
Q08: The presence of parents adds emotional burden on staff.	0.90	0.74–0.96
Q09: Staff experience increased stress due to the presence of parents.	0.89	0.73–0.96
Q10: Staff workload increases when parents are present.	0.80	0.49–0.92
Q11: Staff coordination improves during clinical actions when parents are present.	0.76	0.39–0.91
Q12: Parents may believe the resuscitation team was ineffective.	0.86	0.64–0.94
Q13: Parents may perceive the resuscitation process as chaotic.	0.87	0.67–0.95
Q14: Parents may interfere and hinder the resuscitation process.	0.92	0.79–0.97
Q15: Parental presence during resuscitation may trigger legal action.	0.96	0.91–0.99
Q16: I support parental presence during resuscitation.	0.89	0.73–0.96
Q17: I support parental presence during initial interventions such as tactile stimulation and ventilation.	0.86	0.65–0.95
Q18: I support parental presence during extended resuscitation requiring chest compressions, intubation, and drug administration.	0.83	0.58–0.93

## Data Availability

The data presented in this study are available on request from the corresponding author (the data are not publicly available due to privacy or ethical restrictions).

## Public Involvement Statement

No public involvement in any aspect of this research.

## Guidelines and Standards Statement

This manuscript was drafted against the Consensus-Based Checklist for Reporting of Survey Studies (CROSS) guidelines for survey research [63].
